# Mobility of Nucleostemin in Live Cells Is Specifically Related to Transcription Inhibition by Actinomycin D and GTP-Binding Motif

**DOI:** 10.3390/ijms22158293

**Published:** 2021-08-02

**Authors:** Chan-Gi Pack, Keehoon Jung, Bjorn Paulson, Jun Ki Kim

**Affiliations:** 1 Asan Institute for Life Science, Asan Medical Center, Seoul 05505, Korea; changipack@amc.seoul.kr (C.-G.P.); bjorn.paulson+mtrls@gmail.com (B.P.); 2 Department of Convergence Medicine, University of Ulsan College of Medicine, Seoul 05505, Korea; 3 Department of Anatomy and Cell Biology, Seoul National University College of Medicine, Seoul 03082, Korea; keehoon.jung@snu.ac.kr; 4 Department of Biomedical Sciences, Seoul National University College of Medicine, Seoul 03082, Korea; 5 Institute of Allergy and Clinical Immunology, Seoul National University Medical Research Center, Seoul 03082, Korea

**Keywords:** fluorescence correlation spectroscopy, diffusion coefficient, nucleostemin, nuclear diffusion, ActD, DRB, TSA

## Abstract

In vertebrates, nucleostemin (NS) is an important marker of proliferation in several types of stem and cancer cells, and it can also interact with the tumor-suppressing transcription factor p53. In the present study, the intra-nuclear diffusional dynamics of native NS tagged with GFP and two GFP-tagged NS mutants with deleted guanosine triphosphate (GTP)-binding domains were analyzed by fluorescence correlation spectroscopy. Free and slow binding diffusion coefficients were evaluated, either under normal culture conditions or under treatment with specific cellular proliferation inhibitors actinomycin D (ActD), 5,6-dichloro-1-beta-D-ribofuranosylbenzimidazole (DRB), or trichostatin A (TSA). When treated with ActD, the fractional ratio of the slow diffusion was significantly decreased in the nucleoplasm. The decrease was proportional to ActD treatment duration. In contrast, DRB or TSA treatment did not affect NS diffusion. Interestingly, it was also found that the rate of diffusion of two NS mutants increased significantly even under normal conditions. These results suggest that the mobility of NS in the nucleoplasm is related to the initiation of DNA or RNA replication, and that the GTP-binding motif is also related to the large change of mobility.

## 1. Introduction

Nucleostemin (NS) is a protein that is preferentially expressed in certain vertebrate stem cells and tumor cells, where it is thought to play an essential role in the regulation of stem cell proliferation, self-renewal, and differentiation. While nucleostemin was initially identified as a nucleolar protein, it has long been known to shuttle between the nucleolus and nucleoplasm [1,2], and is therefore apparently involved in cell cycle regulation rather than ribosome production [3]. The shuttling behavior has been attributed to a series of important motifs that make up the structure of NS: a basic motif, a closed-coil motif, two guanosine triphosphate (GTP)-binding motifs (termed G1 and G4), an intermediate motif, and acidic domains [2]. Recent studies have advanced the hypothesis that, instead of promoting cellular growth via nucleolar interactions, the primary role of nucleostemin seems to be tied to DNA replication in the nucleoplasm during S-phase. There, it may coordinate the correction of deleterious mutations that would limit the efficiency of stem (and tumor) cell proliferation [4]. However, a wealth of previously unknown potential interactions between NS and other proteins have been catalogued, including interactions with p53 [5], the alpha isoform of human protein phosphatase 2 regulatory subunit B (PPP2R5A) [6], RSL1D1 [7], telomeric repeat-binding factor 1 (TERF1) [8,9], and mouse double minute 2 (MDM2), which is an E3 ubiquitin-protein ligase [10,11,12]. Meanwhile, other studies have suggested that NS fulfills three distinct roles in the nucleoplasm [4].

In a previous study that conducted fluorescence recovery after photobleaching (FRAP) analysis on NS-expressing cells tagged with (GFP), NS could function in both the nucleolus and nucleoplasm while shuttling between the nucleolus and the nucleoplasm in a GTP-dependent manner [2]. However, the study discussed only the shuttling into and out of the nucleolus due to the technical characteristics of FRAP, which can only analyze the fluorescence of highly expressed NS-GFP, which is mainly localized in the nucleolus. Since it has been suggested that NS interacts with various proteins in the nucleus, it is predicted that specific changes in diffusion patterns may be detected if the diffusion movement in the nucleoplasm is scrutinized, where NS-GFP expression (i.e., fluorescence intensity) is relatively low.

Confocal laser scanning microscopy (CLSM)-based fluorescence correlation spectroscopy (FCS) is one of the most useful techniques to evaluate the concentration, diffusion coefficient (*D*), and interaction of fluorescent molecules at the single-molecule level in live cells as well as in aqueous solution [13,14,15,16]. In the present study, we adopted highly sensitive fluorescence correlation spectroscopy (FCS) to investigate the effect of the replication/transcription inhibitors actinomycin D (ActD), 5,6-dichloro-1-beta-D-ribofuranosylbenzimidazole (DRB), and trichostatin A (TSA) on the diffusion dynamics of GFP-tagged nucleostemin in live cells, with a particular focus on the dynamics of the nucleolar protein in the nucleoplasm. Unlike in cell lyses, diffusion of nucleostemin in the nucleoplasm is observed by FCS to partition into a slow component and a fast component, for the first time to the best knowledge of the authors, and this original observation cannot be explained by the molecular weight of NS alone. It is demonstrated that the fractional ratio of the slow component is reduced when cells are treated with ActD and either the fractional ratio of the slow component is reduced or the slow component is sped up when NS with GTP-binding domain deletions are introduced. This suggests that there is an unknown molecule in the nucleoplasm which restricts NS motility, and that the existence of this compound may be related to DNA or RNA replication or transcription. Our study demonstrates the utility of high-resolution FCS for the investigation of intranuclear binding domains of NS. Determination of the NS-slowing molecule could contribute to the development of more precise therapeutic and diagnostic biomarkers based on NS and its associated proteins.

## 2. Results

### 2.1. FCS Analysis of NS-GFP Diffusion in Aqueous Solution and in Live Cells

**Figure 1:**
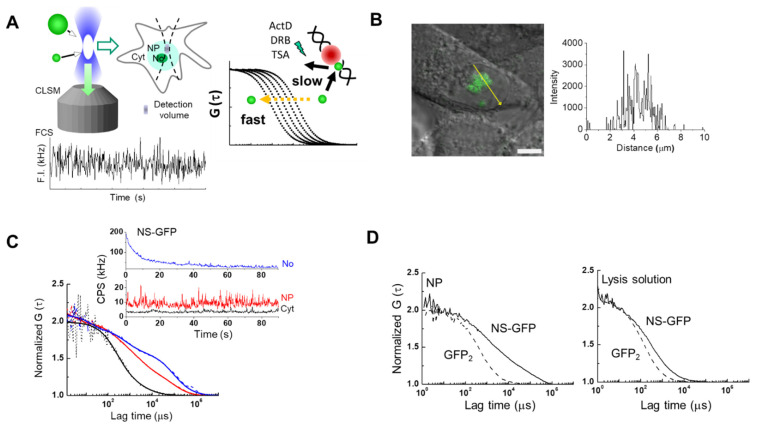
The diffusion rate of GFP-tagged nucleostemin (NS-GFP) expressed in HeLa cells. (**A**) Schematic of experimental workflow. Living single cells were imaged using CLSM to observe cellular localization, followed by FCS measurements to evaluate the diffusion coefficient of the fluorescent molecules (left). Unlike the fast diffusion of molecules in solution, which is determined only by molecular weight and viscosity, the diffusion of intracellular molecules can be slowed by interactions, including the formation of complexes or interactions with organelles. This slow diffusion can be accelerated by certain drugs that inhibit the specific interactions (right). (**B**) Representative confocal micrograph of HeLa cells expressing NS-GFP (left), and the fluorescence intensity along a line crossing the nucleolus and nucleoplasm (right). Scale bar = 5 µm. (**C**) Representative averaged fluorescence intensity (upper) and fluorescence autocorrelation functions (FAFs; G (τ)s, lower) obtained by the FCS measurement of NS-GFP in the cytoplasm (Cyt, black), nucleoplasm (NP, red), and nucleolus (No, blue) of HeLa cells. (**D**) Comparison of FAFs obtained from NS-GFP (89 kDa) and tandemic GFP dimers (60 kDa) in the nucleoplasm (left) and in a lysed sample (right). GFP_2_: dimeric GFP.

Figure 1 shows a schematic of the workflow used to analyze the movement of NS tagged with GFP (NS-GFP) in the cytoplasm, nucleoplasm and nucleoli, using CLSM and FCS (Figure 1A), and to quantitate GFP signals in HeLa cells expressing NS-GFP or dimeric GFP (Figure 1B–D). NS-GFP was mainly located in the nucleolus of HeLa cells, which is in line with previous studies, as shown in Figure 1B [2]. When applied to specific points (i.e., the confocal detection volume of approximately 0.12 fL) in the CLSM image, FCS measures averaged fluorescence intensity in counts per second (CPS in kHz), which varies over time due to random fluctuations in the number of fluorophores (Figure 1C). The averaged fluorescence intensity obtained by FCS measurement is proportional to the fluorescence intensity distribution in each compartment (Figure 1B,C). In the case of the nucleolus, the average fluorescence intensity is rapidly photobleached, decreasing to a steady state over time. In the present study, most of the NS-GFP molecules in the nucleolus were stationary, or the diffusion rate was too slow to avoid photobleaching. In contrast, measurements in the nucleoplasm and cytoplasm could be obtained without photobleaching, indicating that most of the molecules were diffusing freely (inset, Figure 1C and Figure S1). Fluorescence autocorrelation functions [FAFs, G (τ)s] are calculated from the raw intensity data and normalized to the fluorescence intensity, and measure the diffusion of the fluorescent protein at the measured point (Figure S1) [13,17]. In HeLa cells expressing NS-GFP, diffusion of NS-GFP in the dense nucleolus was significantly slower than in the nucleoplasm, which was in turn slower than the diffusion in the cytosol (Figure 1C). Each FAF fits to a two-component model: one fast diffusional component and another slow diffusional component. The fast component represents free diffusion, while the slow component indicates diffusion originating from binding interactions with much larger molecules or immobile structures, such as cellular organelles, although it is not possible to specify what causes such interactions to occur. When the diffusion motion of two or more components is detected, the ratio of the number of fluorescent molecules to each diffusion component can also be evaluated. In live cells, proteins can exhibit various diffusional movements because they can form protein complexes, or interact with organelles, and measurements are complicated by cytoplasmic and nucleoplasmic viscosity [17,18,19]. Therefore, to simplify this complexity, it is helpful to also quantify the intracellular diffusion motion in samples obtained by cell lysis (Figure 1D). 

Figure 1D shows the re-analysis by FCS of an intact sample of HeLa cells expressing dimeric GFP and NS-GFP, as well as re-analysis of a lysed sample of the same. For the fast component of NS-GFP, diffusion was detected to be 3.0 times slower in cytoplasm and 4.4 times slower nucleoplasm than in solution (Table 1). Unlike the GFP dimer, which was detected in the solution sample as a single diffusion component corresponding to its molecular weight (60 kD). Diffusion components for the NS-GFP in solution may be connected to molecular weights via the Stokes-Einstein relation [17], and corresponded to molecular weights of 133 kD and 62 MD, which are slightly larger and much larger, respectively, than the calculated molecular weight of NS-GFP (89 kD). In the cases of the G256V-GFP mutant protein and of the well-known nucleolar protein GFP-B23 (63 kD), diffusion in solution was detected as a single diffusion component corresponding to a molecular weight of 60 kD and 320 kD, respectively (Table 2). 

### 2.2. Localization and Diffusion of NS-GFP before and after Inhibitor Treatment 

**Figure 2:**
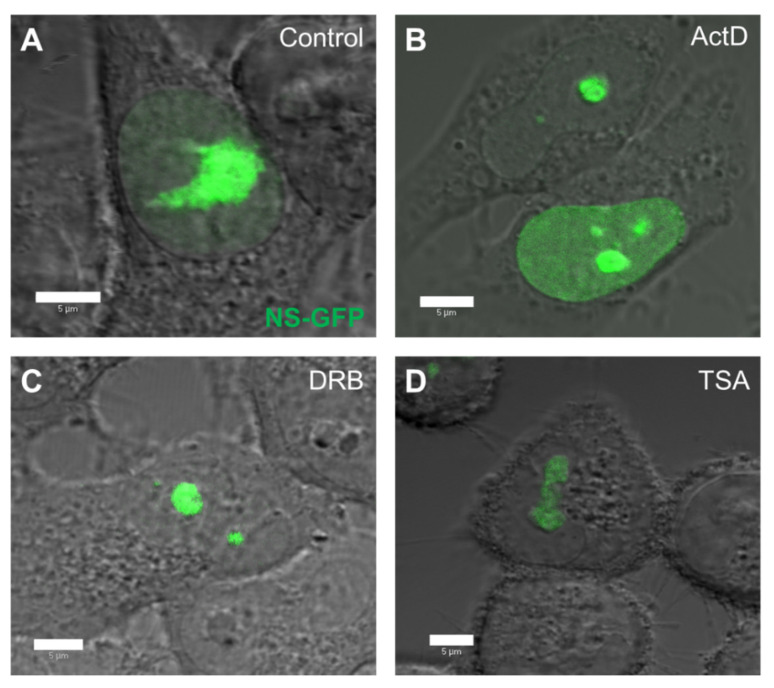
Effect of replication and transcription inhibitors on the nuclear distribution of nucleostemin-GFP (NS-GFP) in HeLa cells. (**A**) HeLa cells expressing NS-GFP imaged by confocal microscopy prior to treatment. (**B**) NS-GFP-expressing HeLa cells after treatment with the DNA replication inhibitor ActD for 3 h. (**C**) NS-GFP-expressing HeLa cells after treatment with the transcription inhibitor DRB for 3 h. (**D**) NS-GFP-expressing HeLa cells after treatment with the chromatin remodeling inhibitor TSA for 3 h prior to imaging. Scale bars = 5 µm.

To quantitatively evaluate the effect of replication and transcription inhibitors on the intranuclear diffusion of NS-GFP, the inhibitors ActD, DRB, and TSA were added to NS-GFP-expressing cells and the diffusion was analyzed by CLSM and FCS [17,18]. Prior to FCS analysis, the fluorescence intensity and localization of NS-GFP was assessed in HeLa cells treated with ActD, DRB, and TSA (Figure 2). ActD is a common chemotherapeutic drug that inhibits transcriptional initiation by intercalating into DNA, inhibiting the progression of DNA replication [20,21]. DRB also inhibits transcription, but does so by blocking RNA polymerase II elongation [22,23], while TSA inhibits chromatin remodeling, and thereby enhances transcription rates [17,24]. Compared to untreated cells, which express NS-GFP primarily in the large nucleolus and weakly in the nucleoplasm, cells treated with ActD had higher levels of NP fluorescence in the nucleoplasm. Cells treated with DRB had reduced NS fluorescence in the nucleoplasm, with NS being highly localized in the nucleolus, while cells treated with TSA showed lower fluorescence intensity of NS-GFP, which was localized in the nucleolus and its immediate proximity.

**Figure 3:**
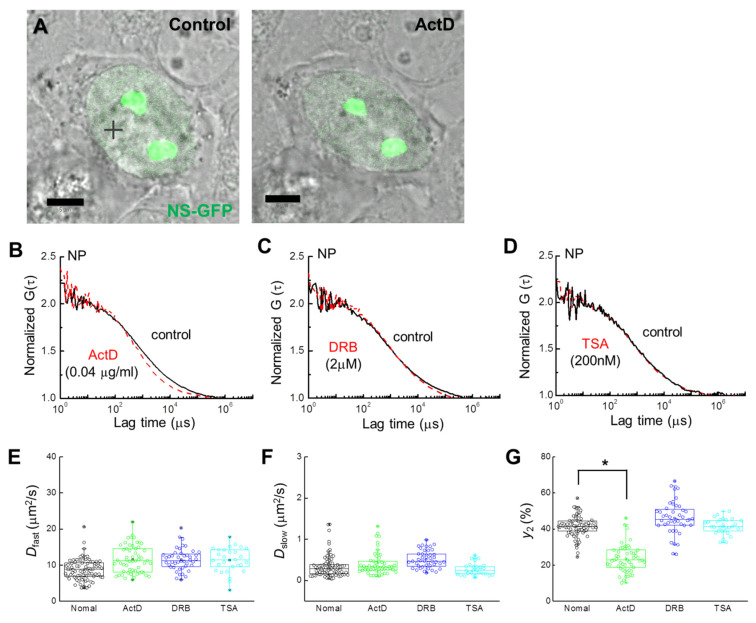
Single-cell analysis of NS-GFP mobility with inhibitor treatment. (**A**) Merged images of CLSM and bright-field microscopy of a HeLa cell expressing NS-GFP (left) before and (right) after ActD treatment. Scale bars = 5 µm. (**B**) Representative FAF [G(τ)] at a position of the nucleoplasm (+) of the HeLa cell in panel (**A**) before (solid black line) and after (dashed red line) treatment with ActD. (**C**) Representative FAFs before (solid black line) and after (dashed red line) DRB treatment (2 μM). (**D**) Representative FAFs before (solid black line) and after (dashed red line) TSA treatment (200 nM). FAFs were normalized to 2 for mobility comparison. Measured FAF for treatment and control groups are represented by dashed red and solid black lines, respectively. For clarity, fit curves are not shown. (**E**–**G**) Box plots showing the distribution across FCS measurements of (**E**) diffusion coefficients for the fast dispersion component, *D_fast_*, (**F**) diffusion coefficients for the slow dispersion component, *D_slow_*, (**G**) fractional ratios for the slow component, *y*_2_. * *p* < 0.02.

FCS analysis of single cells expressing NS-GFP was performed to evaluate the effect of each inhibitor on the diffusion dynamics of NS-GFP (Figure 3A). In the nucleoplasm of HeLa cells expressing NS-GFP, NS-GFP diffusion dynamics fit a two-component model before and after treatment with ActD, DRB, and TSA (Figure 3B–D). While 2 µM DRB and 200 nM TSA caused only slight increases in protein mobility, treatment with 0.04 µg mL^−1^ of ActD significantly increased the diffusional mobility of NS in the nucleoplasm, and reduced the bonding fraction (i.e., the slow diffusion component) from 42% to 20%. A mean diffusion coefficient of 11.6 ± 1.8 µm^2^ s^−^^1^ for fast diffusion was obtained for NS-GFP in cells receiving ActD, while the untreated NS-GFP equivalent was 8.7 ± 1.5 µm^2^ s^−^^1^. Mean *D* of 0.41 ± 0.1 µm^2^ s^−1^ for slow diffusion was obtained for NS-GFP in cells after ActD treatment, while the untreated NS-GFP equivalent was 0.34 ± 0.08 µm^2^ s^−1^ (Figure 3E–G, Table 1). Together, these results suggest that ActD induces both less transient bonding and reduced overall bonding of NS-GFP in the nucleoplasm.

### 2.3. Inhibitor Dosage Dependence and Effect on Multimeric GFP and GFP-B23

**Figure 4:**
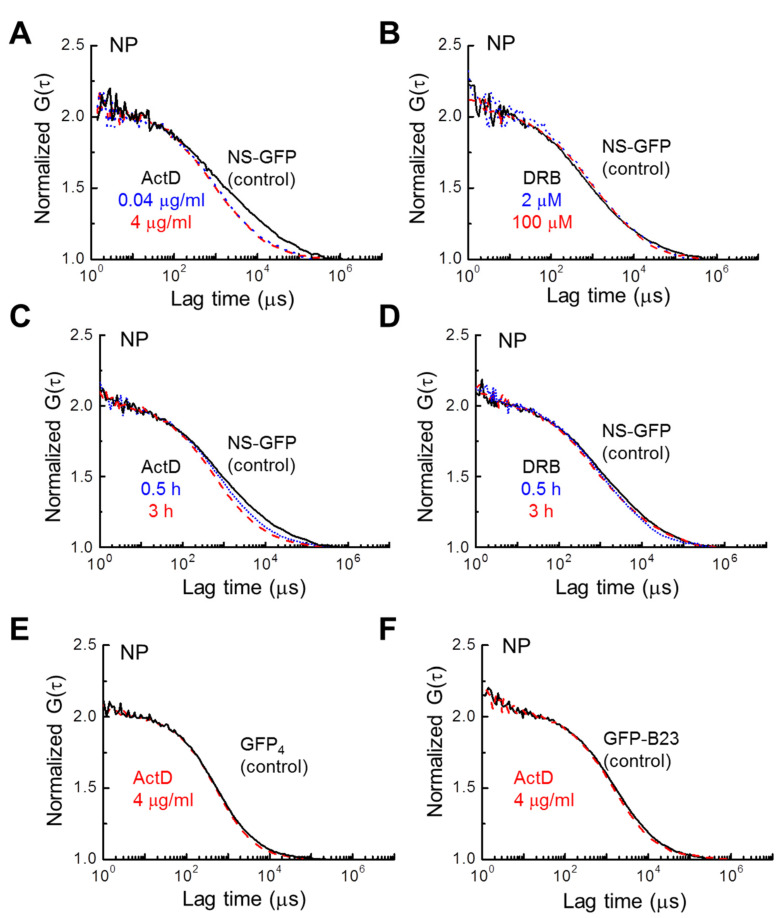
Effect of inhibitor concentration and treatment time on NS-GFP mobility in the nucleoplasm. Representative FAFs before and after treatment with two concentrations of (**A**) ActD and (**B**) DRB. Changes in FAFs with two different treatment durations for (**C**) ActD and (**D**) DRB. (**E**) Representative FAFs measured in the nucleoplasm of HeLa cells expressing tetrameric GFP (indicated by GFP_4_), and (**F**) GFP-B23 before and after ActD treatment. To compare mobilities, each FAF was normalized to a value of 2. In all frames, control samples are represented by solid black lines, while dotted blue lines indicate the low-concentration or short-duration treatment groups, and dashed red lines represent high-concentration or long-duration treatment groups.

The dose-dependent effect of ActD on NS-GFP mobility may provide more insight into the nuclear dynamics of NS. The fluorescence correlation functions of NS-GFP with dosage and treatment time were calculated for the replication/transcription inhibitors ActD and DRB (Figure 4). Increasing ActD dosage from 0.04 µg mL^−1^ to 4 µg mL^−1^ resulted in no additional appreciable change in the diffusion coefficient (Figure 4A). Likewise, increasing DRB dosage from 2 µM to 100 µM did not change the diffusion dynamics, nor was the DRB treatment duration significant (Figure 4B,D). However, an increasing diffusion coefficient was correlated to increasing ActD treatment duration (Figure 4C). To determine whether this effect was due to the inherent dynamics of the NS binding motifs, or due to the incorporation of GFP, the same experiment was repeated with HeLa cells expressing tetrameric GFP. In addition, for comparison with NS, another well-known nucleolar protein, B23 tagged with GFP (GFP-B23), was analyzed to see if the same change in the diffusion coefficient occurred with ActD treatment [25,26,27]. In contrast to NS-GFP, no changes in the diffusional dynamics were detected for these tetrameric GFP and GFP-B23 proteins with ActD treatment, indicating that the ActD treatment increases the diffusion speed of NS-GFP due to a reduction in NS bonding, rather than GFP, and that the effect is unique to the nucleolar protein NS (Figure 4E,F). On the other hand, when compared to GFP-Rpb I (RNA polymerase II tagged with GFP), significant changes in diffusional mobility were observed for both ActD and DRB treatments (Figure S2A,B). 

**Figure 5:**
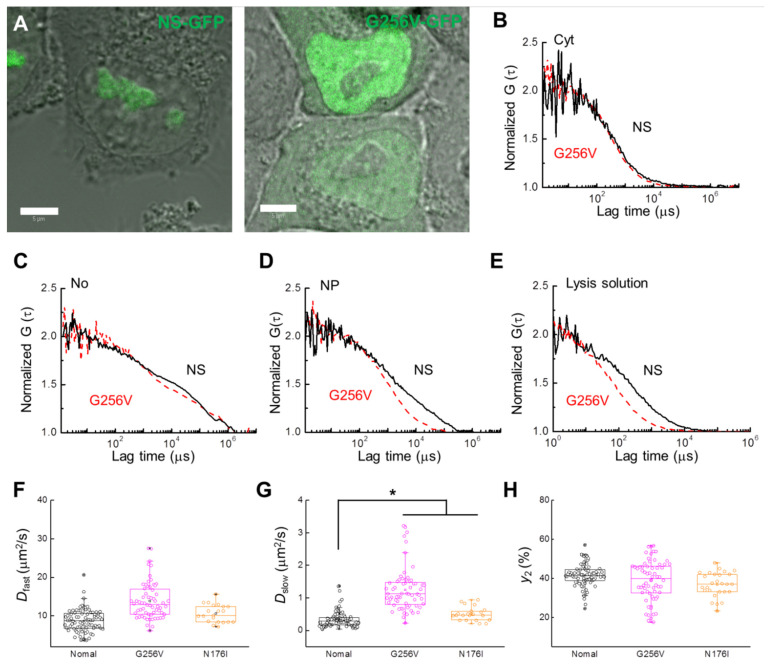
Comparison of NS-GFP mobility with GTP-binding motif NS mutant G256V-GFP in live HeLa cells and lysed cell solution. (**A**) Merged CLSM and bright-field images of HeLa cells expressing (left) NS-GFP and (right) G256V-GFP. Scale bars = 5 µm. (**B**) Representative FAFs [G(τ)] obtained from NS-GFP (solid black line) and G256V-GFP (dashed red line) in the cytosol (Cyt), (**C**) the nucleolus (No), and (**D**) the nucleoplasm (NP) of HeLa cells. (**E**) FAF obtained from the supernatant solutions of lysed cells expressing each protein. To compare mobility between samples, each FAF was normalized to a value of 2. Box plots show the distribution of values across measurements for the (**F**) diffusion coefficients of the fast diffusion component, *D_fast_*, (**G**) diffusion coefficients for the slow dispersion component, *D_slow_*, (**H**) fractional ratios for the slow component, y_2_. * *p* < 0.02.

There are two GTP motifs on NS, and these motifs are closely related to the nucleolus-nucleoplasm shuttling function of NS [2]. In this study, FCS analysis was performed on the mutant proteins G256V and N176I, tagged with GFP, to determine whether these GTP motifs also affect the slow diffusion component detected in the nucleoplasm. HeLa cells expressing the G256V and N176I single amino acid substitutions in the G1 and G4 GTP-binding motifs of NS-GFP, respectively, were investigated by CLSM and FCS. Compared to the GTP-bound wild type NS, G256V-expressing HeLa cells showed significantly more relative fluorescence in the nucleoplasm, with even more nucleoplasmic localization than nucleolar localization (Figure 5A). Interestingly, while NS diffusion was unaffected by the G256V mutation in the nucleolus or in the cytosol, the G256V mutant showed a significant increase in diffusion mobility in the nucleoplasm and in lysed solutions of the cells (Figure 5B–E, see also Table 1 and Table 2). Unlike the effect of the ActD treatment, in which the ratio of the slow diffusion component was largely reduced, in the G256V mutant the diffusion coefficients of the slow diffusion components increased (Figure 5F–H, Table 1). On the other hand, N176I showed strong localization in the nucleolus, similar to NS, but its diffusion rate in the nucleoplasm was greatly accelerated compared with that in the nucleolus (Figure S3 and Table S1). However, mobility in the nucleoplasm was less changed compared to G256V (Figure 5F–G).

## 3. Discussion

It has previously been shown that the mobility of monomeric and multimeric GFP within the nucleolus and nucleoplasm follows a one-component diffusion model (*i* = 1) [18]. In validation of previous results, a single diffusion component was obtained for dimeric GFP, with observed reduction in diffusion coefficients by factor of 3.5 in nucleoplasm, in good agreement with previous demonstrations of viscous diffusion of such molecules in the cytoplasm and the nucleus [17,18,19]. 

In contrast to the single-component diffusion of GFP, in the present study, the mobility of NS-GFP fitted a two-component model (*i* = 2), in which one species of molecule diffuses freely through the nucleoplasm, while another species is slowed by dynamically binding to immobile (or very slowly moving) nuclear structures, such as chromatin. As expected of free diffusion, NS and its derivatives in lysed solutions approximately maintained the cubic relationship between diffusion coefficient and molecular weight predicted by the Stokes-Einstein relation [17], but this idealized relationship broke down in measurements of the slow diffusion of NS-GFP in the nucleoplasm and nucleolus. Considering the viscosity in the cell nucleoplasm, the two components of NS-GFP diffusion detected in lysis solution appear to be the same two components detected in the intact cells (Table 1 and Table 2). In particular, the fast component of NS-GFP in the cell is consistent with a factor of four decrease in mobility from solution due to the increased nuclear viscosity, while the slow component shows an anomalously higher (14×) decrease in diffusion coefficient. On the other hand, for the other protein factors for which only one diffusion component was detected in lysed solution, the fraction that interacts with the unknown component and the fraction that freely diffuses can coexist in the nucleoplasm, so they appear to be partitioned into slow and fast-diffusing components. In the lysed sample, only one component of the diffusion coefficient of G256V-GFP was detected, and the diffusion coefficient approximately corresponded to the calculated molecular weight of G256V-GFP. Therefore, it can be presumed that G256V rapidly diffuses in the nucleoplasm of cells because it does not form a large complex, unlike NS.

NS-GFP showed some effect in the modulation of NS-GFP fluorescence intensities. Cells treated with ActD had higher levels of NP fluorescence in the nucleoplasm than untreated cells, possibly due to nucleolar reorganization, which has previously been attributed to degradation of nucleolar structure under ActD treatment [2,18,25,26]. 

ActD is well known to inhibit the initiation of DNA or RNA replication, and DRB is also known to inhibit transcriptional elongation. Therefore, it seems that both inhibitors rapidly changed the intranuclear motility of GFP-RpbI, while they had no effect on multimeric GFP or GFP-B23 independent of transcription factors. The fraction of bound NS-GFP in the nucleoplasm did not vary after DRB treatment, but varied from 42% before ActD treatment to 20% after ActD treatment. 

A few technical challenges may limit the specificity of fraction measurements in the present study. The confocal volume may limit the specificity of the fraction measurement if the confocal volume extends past the imaging domain. For this reason, measurements were limited to cellular compartments greater than twice the largest dimension of the confocal volume in diameter [17,28]. Photobleaching may also appear in the data as an additional very slow diffusion component if not controlled, but it was only observed in the nucleolus, and was corrected for by an initial bleaching step (Figure S1). The potential case of nonuniform bleaching rate between populations was not specifically controlled for. Since the fraction ratios for the slow diffusion component are relatively large, while the observed fluorescence intensity change over each measurement is very small, bleaching is not a satisfactory explanation for the results as a whole.

The observation of ActD modulation of the NS-GFP slow diffusion coefficient indicates that ActD potentially induces both less transient bonding and reduced overall bonding of NS-GFP in the nucleoplasm. Furthermore, the fact that the diffusion of NS-GFP is accelerated only by ActD, which is an inhibitor of the initiation of DNA replication, strongly suggests that NS-GFP is involved in the formation of a series of complexes related to the initiation of DNA replication. Although it is unclear what molecular interactions maintain the slow diffusion of NS-GFP, our results thus suggest that NS-GFP is closely related to the initiation of DNA or RNA replication. 

**Figure 6:**
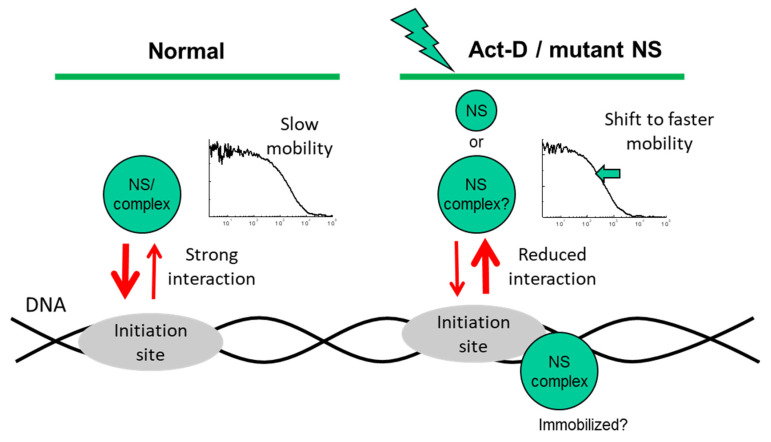
Proposed mechanism of potential interactions with an unknown target, as derived from the observed nuclear mobility of nucleostemin (NS-GFP) in the present study.

Consequently, our results show that diffusion of NS in the nucleoplasm can be accelerated by treatment with ActD, or by disabling the G1 or G4 GTP-binding domain (Figure 6). Although it is unclear why the diffusion coefficients of NS-GFP and G256V-GFP in solution differ greatly, the binding effect expressed by the slow diffusional components of NS-GFP and mutants in the nucleoplasm is unique to the intranuclear environment, suggesting mechanisms such as transcriptional activity. 

Previous studies have proposed a model in which NS in the nucleoplasm binds to MDM2 (mouse double minute 2), an E3 ubiquitin ligase that promotes the degradation of p53 [4,10]. ActD treatment decreases MDM2 levels at both low and high concentrations [29]. This model suggests that the diffusion of NS-GFP in the nucleoplasm is enhanced under ActD treatment due to reduced immobilization by MDM2. However, binding MDM2 to nucleostemin involves the coiled-coil and acidic domains of NS [11,12], rather than the GTP domains, whose mutation abrogated bonding in the present study. Although it is possible that the GTP domain and the observed ActD-dependent increase in mobility are the result of different causal mechanisms, more evidence is needed to elucidate these mechanisms.

ActD may have compounding effects in modulating the effect of GTP-binding NS mutants. While the changes in the intranuclear diffusion of GTP-binding mutants even under non-treatment conditions are approximately the same in magnitude as those changes which occur in NS diffusion after ActD treatment, as shown in Figure 4 and Figure 5, these may derive from different root causes, as the fractional ratios are observed to differ in Table 1. Thus, potential compounding effects and linkages between the GTP-binding NS mutants and ActD may deserve further consideration in follow-up studies.

While this paper has demonstrated use of the high-sensitivity FCS method to identify potential interactions between NS and molecules in the nucleoplasm which are inaccessible to conventional molecular biology techniques, clarification of the full range of potential bonding targets of NS and elucidation of these targets by additional techniques would clarify and solidify the results of this research. Such work may include assessing the role of NS in transcription initiation or the mobility of NS in different phases of the cell cycle. While this is beyond the scope of the present study, in addition to MDM2 there are several alternative candidates that may be responsible for the observed slow diffusion. 

In particular, there are several alternative candidate proteins that are known to interact with NS in the nucleoplasm, namely telomeric repeat binding factor 1 (TRF1/TERF1) and promyelocytic leukemia protein isoform IV (PML-IV) [30], RAD51 [31,32], PPP2R5A [6], and RSL1D1 [7]. Only TERF1 is known to be a mediator of transcription by RNA polymerases [33,34], and hence is a good potential candidate for ActD inhibition, while ActD is not known to have effects on Rad51 [35], PPP2R5A, RSL1D1, or PML-IV. 

## 4. Materials and Methods

### 4.1. Inhibitors

All the following commercially available chemical reagents were used without further purification: ActD (Sigma-Aldrich, St. Louis, MO, USA), DRB (Sigma-Aldrich), and TSA (Sigma-Aldrich). The concentration of each inhibitor used is described in the figure legend or as a note below the relevant table.

### 4.2. Plasmids

Nucleostemin-pEGFP-N1, G256V-pEGFP-N1, N176I-pEGFP-N1, pEGFP-B23-C1, GFP-RpbI, and dimeric GFP (GFP_2_) and tetrameric GFP (GFP_4_) were used to express NS, G256V, N176I, B23, RpbI tagged with GFP, and multimeric GFPs, respectively, as described in previous studies [2,17,18,36]. Plasmid constructs were verified by sequencing. All of the plasmid constructs used for transfection were purified using a plasmid DNA Midiprep kit (Qiagen, Hilden, Germany), and were verified by sequencing.

### 4.3. Cell Culture and Treatments

HeLa cells were grown in a 5% CO_2_ humidified atmosphere at 37 °C in an eight-well chambered coverglass (Nunc, Roskilde, Denmark) for confocal imaging and FCS measurement. Culture medium was Dulbecco’s modified Eagle’s medium (DMEM; Sigma-Aldrich) supplemented with 10% FBS, 100 U mL^−1^ penicillin, and 10 mg mL^−1^ streptomycin. HeLa cells were transiently transfected with the above plasmid vectors. The plasmids (100 ng each) were transfected using FuGENE^®^ HD Transfection Reagent (Promega, Madison, WI, USA), followed by selection with the relevant antibiotics. Aliquots of ActD, DRB, and TSA solutions were added directly to the cell culture medium. Treated cells were dosed with either ActD (0.04 or 4 µg mL^−1^), DRB (2 or 100 µM), or TSA (200 nM), as specified in the text and figure labels. Cells were cultured for 3 h, except in the case of dosing experiments, during which ActD was administered to separate sample groups for 30 min and for 3 h, and DRB was administered to separate sample groups for 30 min and for 3 h. 

### 4.4. Cell Lysis

Cells expressing NS-GFP, G256V-GFP, dimeric GFP, or GFP-B23 were lysed in detergent solution buffer (CelLytic M, Sigma-Aldrich). The lysed solution was spun down by centrifugation at 100,000 rpm for 20 min and the supernatant solution was measured by FCS [17].

### 4.5. Live Cell Imaging

Live cell imaging was performed using an inverted confocal laser scanning microscope (LSM510; Carl Zeiss, Jena, Germany). Confocal observations were performed at 25 °C. GFP was excited at 488 nm using a CW Ar^+^ laser through a water immersion objective lens (C-Apochromat, 40×, 1.2 NA; Carl Zeiss), with emission detected at 505–550 nm for single scanning experiments using cells expressing GFP and GFP-tagged proteins. The pinhole diameters for confocal imaging were adjusted to 70 mm for GFP.

### 4.6. Fluorescence Correlation Spectroscopy

All FCS measurements were performed at 25 °C on a ConfoCor 2–LSM510 microscope (Carl Zeiss, Jena, Germany), as previously described [37,38,39]. GFP was excited at 488 nm, with power minimized by adjustment of an acousto-optical tunable filter. For collection of GFP emissions, the detection pinhole was fixed at a 70-µm diameter, and the emission spectrum was band-pass filtered to 505 to 550 nm. The FCS setup pinhole, the structure parameter *s*, and the detection volume were calibrated daily against FCS measurements of a 10^−7^ M rhodamine 6G solution, which has a known diffusion constant of 280 μm^2^ s^−1^, as described previously [17,18,19,28,40,41].

FCS measurements of lysed cell samples were captured for 30 s and repeated five times with an interval of 2 s on each of three independently prepared samples, while live cell measurements were captured for 90 s for 1 or 3 repetitions on each of independently prepared samples, using low fluorescent cells at a concentration of <20 molecules per detection volume to minimize the effect of photobleaching during FCS analysis [27]. In the nucleolus, rapid photobleaching may occur. In this case, analysis was performed on the part of the cell where the fluorescence intensity became stationary after bleaching was no longer apparent (Figure S1). The measurement position was chosen on the CLSM image, and a fluorescently tagged cover glass was used to align the absolute measurement position between devices, which resulted in an observed misalignment of <0.5 μm. This misalignment is too small to affect the analysis of diffusion around the nucleolus, the size of which tends to be approximately 2 µm × 5 µm.

Fluorescence correlation functions (FAFs), *G*(*τ*), were derived from experimental measurements using the method of Rigler et al. [13,17], using a time series of the observed fluorescence intensity *I*(*t*) from the detection volume delayed by the time *τ*, as follows:

(1) $$G\left(\tau \right)=\langle I\left(t\right)I\left(t+\tau \right)\rangle /{\langle I(t)\rangle }^{2}.$$

Here, brackets denote ensemble averages. The resultant FAFs were fitted to a one- or two-component free diffusion model with or without a triplet term [17,18]:

(2) $$G\left(\tau \right)=1+\frac{1}{N}\sum_{i}y_{i}{\left(1+\frac{\tau }{\tau_{i}}\right)}^{-1}{\left(1+\frac{\tau }{s^{2}\tau_{i}}\right)}^{-1/2},$$

where *y_i_* is the term fraction and *τ_i_* is the translational diffusion time for the *i-*th component, *N* is the number of fluorescent molecules in the detection volume when the volume is approximated as a cylinder, and *s* is a structure parameter defined as the ratio of the axial length *z*_0_ to the beam waist *w*_0_ (*z*_0*/*_*w*_0_). The structure parameter was verified prior to measurement each day of the experiment, and was stable and equal to five. For simplicity, the equation of the triplet term is not shown, because it is independent of diffusion [42]. For FCS analysis, the average value of the structure parameters remained constant for all data obtained under each measurement condition on each day of the experiment.

The diffusion constant, *D_i_*, was calculated from the diffusion time (*τ_i_*) of the “fast” and “slow” components as follows:

(3) $$D_{i}=\;\omega^{2}/4\tau_{i},$$

where ω is the beam waist.

All FAF values were fitted using the software provided with the ConfoCor 2 system [39] using the model of Equation (2) as described previously [17,18,19,40,41,43]. FAFs measured in samples in solution were fit to a one- or two-component model under the assumption of viscous free diffusion, while correlation functions from cells were fit to a two-component model that captures the dynamics of non-bound fast diffusion and bound or interacting slow diffusion, as described in the previous studies [40,41,43]. Briefly, the two-component model was applied when the fit deviation to a single-component model fit was systematically shifted, indicative of a poor single-component fit [17,28]. By the same criterion, all data were observed to fit to a one- or two-component model. The ratio of the number of fluorescent molecules from the fast and slow components of the two-component model was used to calculate the fraction ratio *y_i_/*(*y_fast_ + y_slow_*), which was represented as a percentage. A method for estimating the molecular weight of a spherical or rod-like molecule from the Stokes-Einstein relation using a standard probe and the diffusion time (*τ*_i_) or diffusion coefficient (*D_i_*) of the target factor was followed in accordance with previous studies [17,40,43].

### 4.7. Statistical Analysis

Student’s *t*-test or one-way analysis of variance (ANOVA) were performed to evaluate the significance of differences in mean value (Origin v.8.5; Northampton, MA, USA), and *p* < 0.02 were considered significant.

## 5. Conclusions

In this study, we were able to discover important clues related to NS function by analyzing the specific molecular motion of NS-GFP in the nucleus using high-sensitivity FCS analysis. The study has demonstrated that the slow motility of NS found in the nucleoplasm is likely caused by binding to an unidentified target molecule or immobile structure, which may be inactivated by ActD treatment. This binding seems to also depend on the GTP-binding motifs of NS. These results suggest that the function of NS is closely related to DNA/RNA replication or transcription factors in the nucleoplasm, although the target counterpart and related mechanism remain unknown. Our study will help to elucidate NS target factors and mechanisms of action related to GTP binding and transcriptional activity in the nucleoplasm in the future.

## Figures and Tables

**Table 1:** Summary of diffusion coefficients (*D*s) and fractional ratios (*y*_1_, *y*_2_) obtained by FCS analysis for each protein expressed in HeLa cells (*n* =15). Mean values with standard deviations are shown. * *p* < 0.02.

Protein	Fast Component		Slow Component	
(Expressed in the Nucleoplasm Unless Specified)	*D* (µm^2^ s^−1^)	*y*_1_ (%)	*D* (µm^2^ s^−1^)	*y*_2_ (%)
NS-GFP	8.7 ± 1.5	58	0.34 ± 0.08 *	42 ± 4 *
NS-GFP in cytosol	12.8 ± 2.2	91	0.06 ± 0.03	9 ± 6
NS-GFP/ActD ^1^	11.6 ± 1.8	80	0.41 ± 0.1	20 ± 3 *
NS-GFP/DRB ^1^	12.2 ± 3.6	55	0.60 ± 0.3	45 ± 6
NS-GFP/TSA ^1^	11.3 ± 3.4	59	0.28 ± 0.2	41 ± 4
G256V-GFP	15.0 ± 2.1	61	1.3 ± 0.3 *	39 ± 5
N176I-GFP	11.0 ± 1.6	64	0.6 ± 0.1 *	36 ± 5
GFP-B23	11.7 ± 3.1	30	3.0 ± 0.5	70 ± 8
Dimeric GFP	14.2 ± 1.9	100	N.D. ^2^	

^1^ Dosage of ActD was 0.04 µg mL^−1^ for 3 h, DRB was dosed at 2 µM for 3 h, and TSA was dosed at 200 nM for 3 h. ^2^ A one-component model was used since only one component of diffusion was observed in the sample. N.D. means not detected.

**Table 2:** Summary of diffusion coefficients in the solutions of lysed cells expressing each protein.

In Lysed Solution	Fast Component (*D*, µm^2^ s^−1^) (*y*_1_, Fraction%)	Slow Component (*D*, µm^2^ s^−1^) (*y*_2_, Fraction%)
NS-GFP	38.0 ± 2.9 (64)	4.9 ± 0.4 (36 ± 5)
G256V-GFP	51.0 ± 2.3 (100)	N.D. ^1^
GFP-B23	28.4 ± 0.7 (100)	N.D. ^1^
Dimeric GFP	49.5 ± 1.0 (100)	N.D. ^1^

^1^ A one-component model was used since only one component of diffusion was observed in these samples, indicative of single molecular species. Mean values are shown for three independent experiments. N.D. means not detected.

## Data Availability

All data is contained within the article.

## Supplementary Materials

The following are available online at https://www.mdpi.com/article/10.3390/ijms22158293/s1, Figure S1: Averaged fluorescence intensity and time range of FCS analysis, Figure S2: FAFs of GFP-RpbI expressed in the nucleoplasm of HeLa cells before and after treatment with ActD and DRB, Figure S3: Comparison between the FAFs of N176I-GFP and NS-GFP in the nucleoplasm and nucleolus.
