# Supplementary material for: Mobility of Nucleostemin in Live Cells Is Specifically Related to Transcription Inhibition by Actinomycin D and GTP-Binding Motif
Source: Int J Mol Sci. 2021 Aug 2;22(15):8293. doi: 10.3390/ijms22158293 (PMC8347349; doi:10.3390/ijms22158293)
Supplement: Supplementary file 1 [file ijms-22-08293-s001.zip › ijms-1212969-supplementary.pdf]

International Journal of
Molecular Sciences

Supplementary information

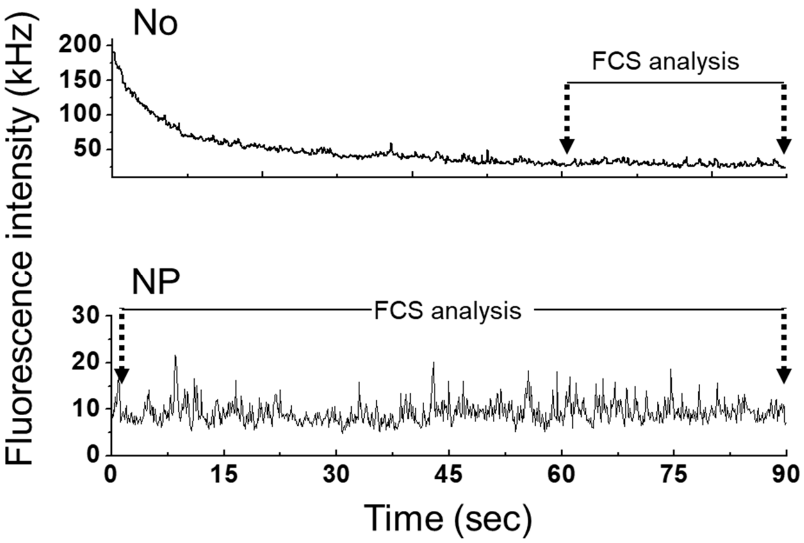

Figure S1. The averaged fluorescence intensity in the nucleolus was high, and photobleached rapidly. Conversely, the averaged fluorescence intensity in the nucleoplasm was low, and photobleaching did not occur. In the nucleolus, FCS analysis was performed to measure the fluorescence intensity after photobleaching was no longer apparent. (bottom) The fluorescence intensity in the nucleoplasm is not limited by photobleaching.

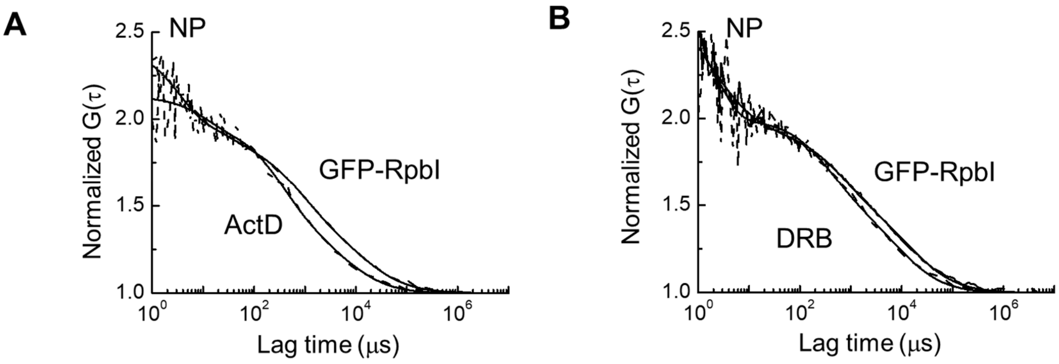

Figure S2. Single-cell analysis of the effect of inhibitor treatment on GFP-RpbI mobility, according to FCS. Representative fluorescence correlation functions of GFP-Rpb I (Pol II) expressed in the nucleoplasm of a single HeLa cell treated with (**a**) ActD and (**b**) DRB.

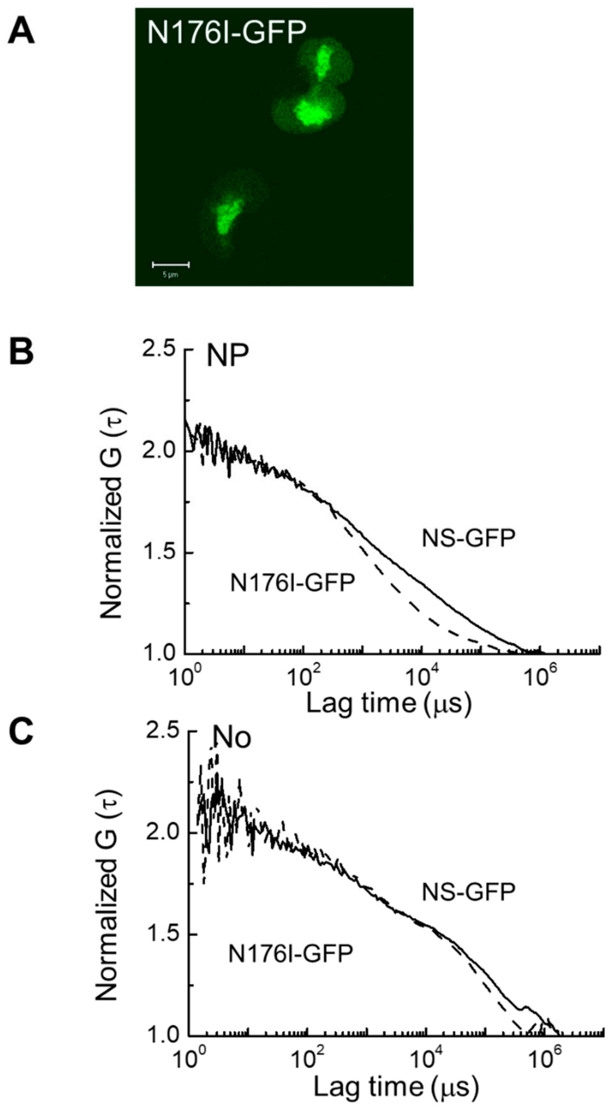

Figure S3. Comparison between the diffusion of N176I-GFP and NS-GFP in HeLa cell. (**a**) Representative confocal micrograph of HeLa cells expressing N176I-GFP, one of the two GTP-binding mutants of NS. (**b**) Diffusion in the nucleoplasm (NP) and (**c**) in the nucleolus (No).
